# Supplementary material for: PATL2 is a key actor of oocyte maturation whose invalidation causes infertility in women and mice
Source: EMBO Mol Med. 2018 Apr 16;10(5):e8515. doi: 10.15252/emmm.201708515 (PMC5938616; doi:10.15252/emmm.201708515)
Supplement: Supplementary file 1 — Appendix [file EMMM-10-e8515-s001.pdf]

APPENDIX

PATL2 is a key actor of oocyte maturation whose invalidation causes infertility in women and mice

Table of contents

SUPPLEMENTARY FIGURES 2

Figure S1. 2

Figure S2. 3

Figure S3. 4

Figure S4 5

Figure S5. 6

Figure S6. 7

Figure S7. 8

Figure S8. 9

Figure S9. 10

SUPPLEMENTARY TABLES 11

Table S1 11

Table S2 12

SUPPLEMENTARY FIGURES

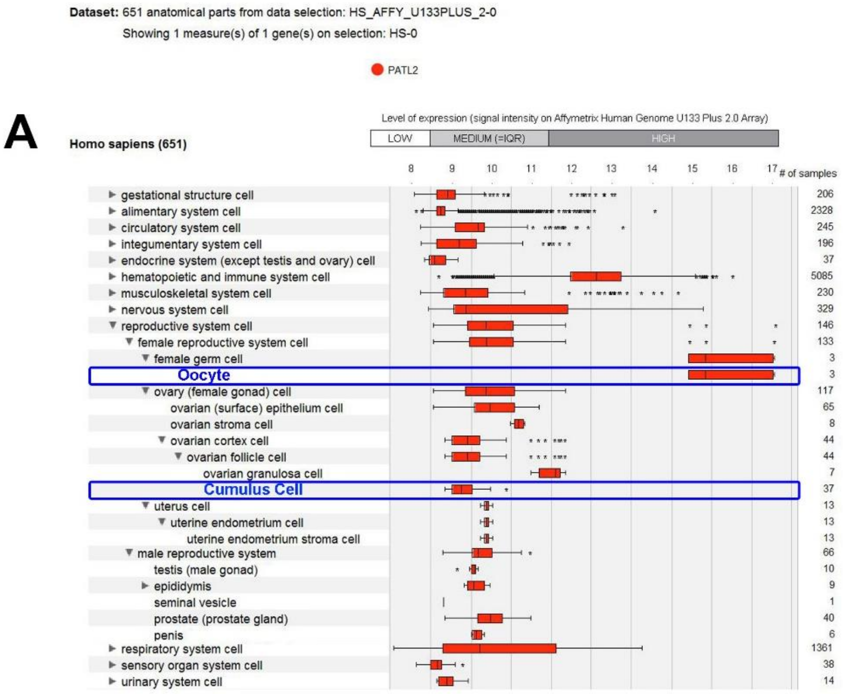

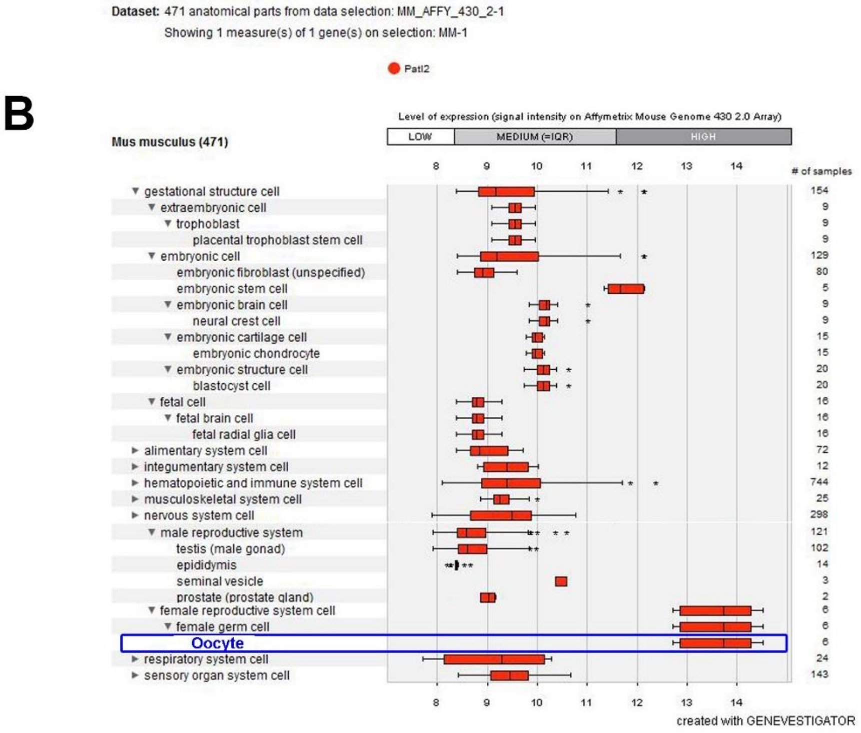

Figure S1. **Relative mRNA expression levels for human and mouse *PATL2* transcripts.**

(A) *PATL2* mRNA levels were measured in different tissues/cells in Humans using Affymetrix microarrays (data available from the Genevestigator database, https://genevestigator.com). Blue rectangles highlight the expression level in oocytes and follicular cells. Oocytes exhibit the highest level of *PATL2* expression, whereas its level is very low in follicular cells. **(B)** Similar data for mice. Data were generated with Genevestigator (Hruz T, Laule O, Szabo G, Wessendorp F, Bleuler S, Oertle L, Widmayer P, Gruissem W and P Zimmermann (2008) Genevestigator V3: a reference expression database for the meta-analysis of transcriptomes. Advances in Bioinformatics 2008, 420747)

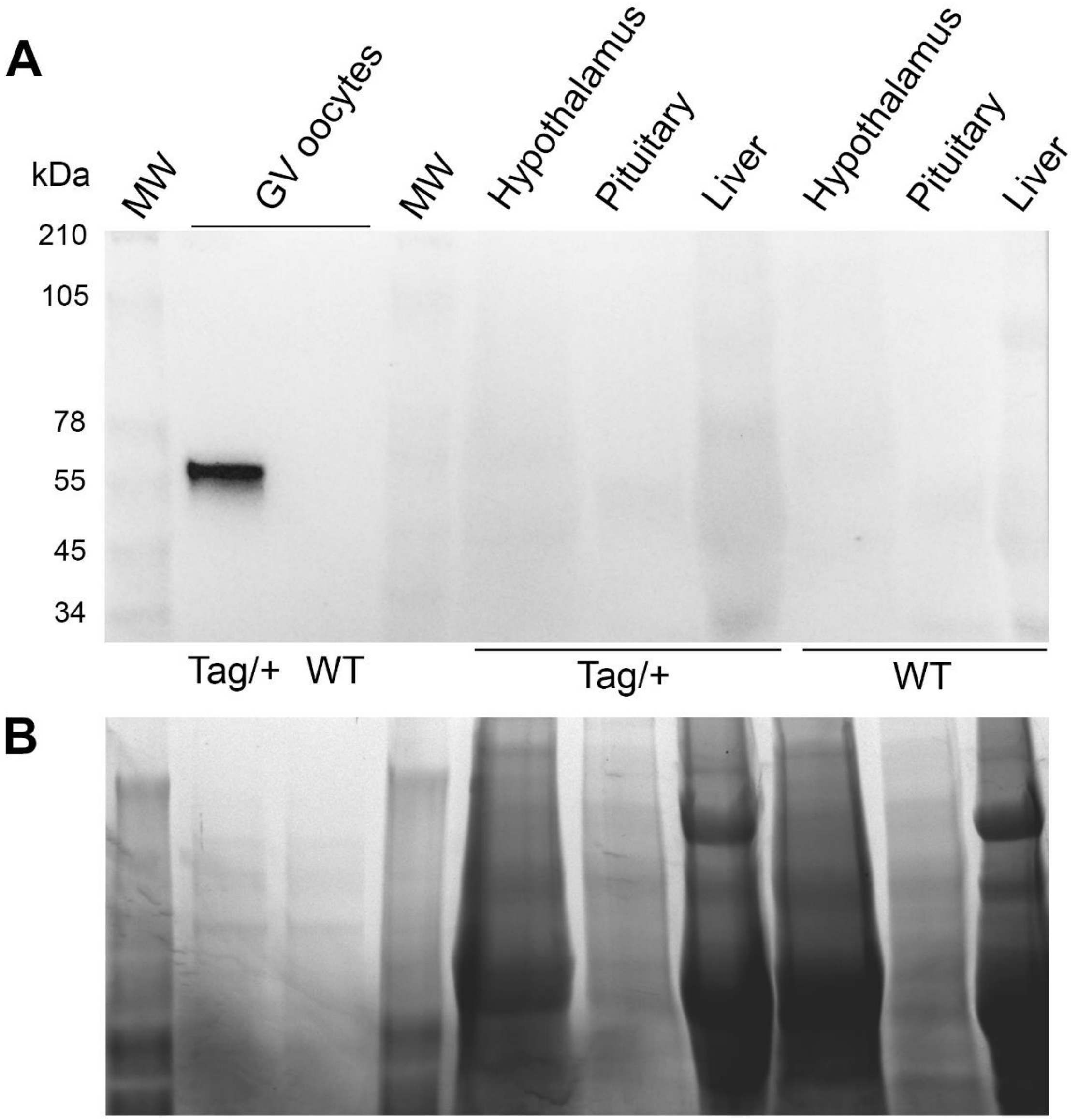

Figure S2. **Western blot comparison of expression of Patl2-HA in oocyte, hypothalamus and pituitary gland extracts.**

(**A**) Western blot of protein extracts from different tissues as indicated and revealed with anti-HA antibodies. Tissues were collected from WT or from Patl2-HA tagged females (tag/+) and 130-140 GV oocytes were loaded per well. Protein extracts were obtained by pooling the organs of 3 different WT or Patl2-HA tagged animals. In the lane loaded with GV oocyte extract from Patl2-HA tag mice, a band at around 60 kDa was observed (expected MW for mouse Patl2-HA = 60.74 KDa), whereas no bands were observed in the other extracts. MW = Molecular Weight (**B**) Protein loads of the Western blot presented in (A) were controlled with TGX stain free™ precast gels.

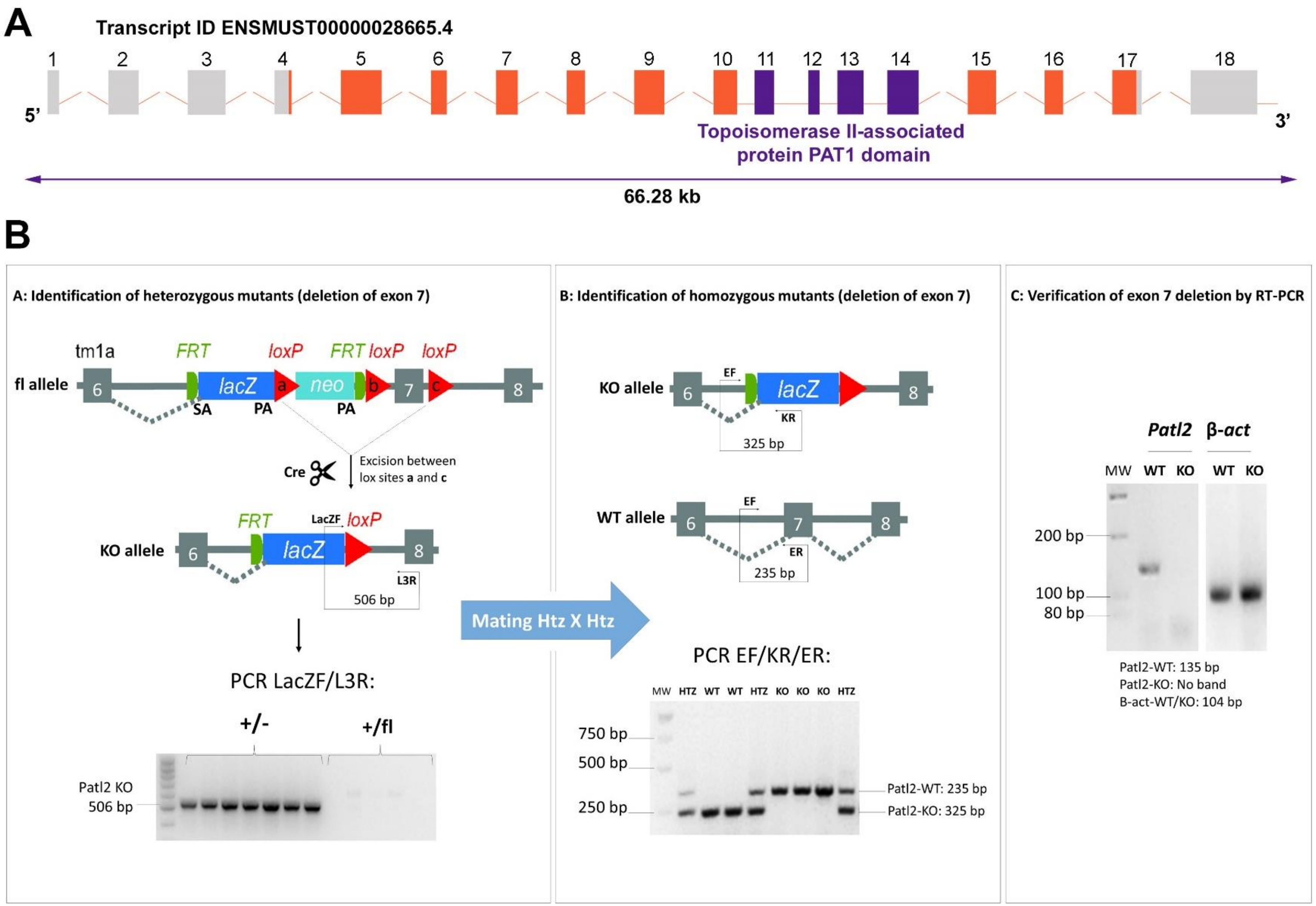

Figure S3. **Genetic modification of *Patl2*-deficient mice and genotyping strategy**

(A) Exon map showing the localization of the topoisomerase II associated Pat1 domain, downstream of exon 7 **(B1)** *Patl2*$^{-/-}$ mice (C57BL/6NTac-Patl2<tm1a) were generated by the EUCOMM Consortium (http://www.mousephenotype.org/about-ikmc/eucomm). The allele map shows the insertion of a LacZ and a neomycin cassette between exons 6 and 7. The critical exon 7 is surrounded by two LoxP sequences. *Patl2*$^{-/-}$ mice were crossed with adult heterozygous EIIaCre transgenic mice which express the CRE-recombinase enzyme ubiquitously from an early stage. Offspring were screened by PCR using LacZF/L3R primers to detect exon 7 deletion. **(B2)** Heterozygous mutant mice showing deletion of exon 7 were mated together and homozygous exon-7-deleted animals were identified by multiplex PCR using EF/KR/ER primers. **(B3)** RT-PCR was carried out on selected F2 animals to verify deletion of exon 7 in *Patl2*$^{-/-}$ animals using primers within exon 7. The band at 135 bp was sequenced and corresponded to *Patl2*. Sequences of all primers are indicated in Table S6.

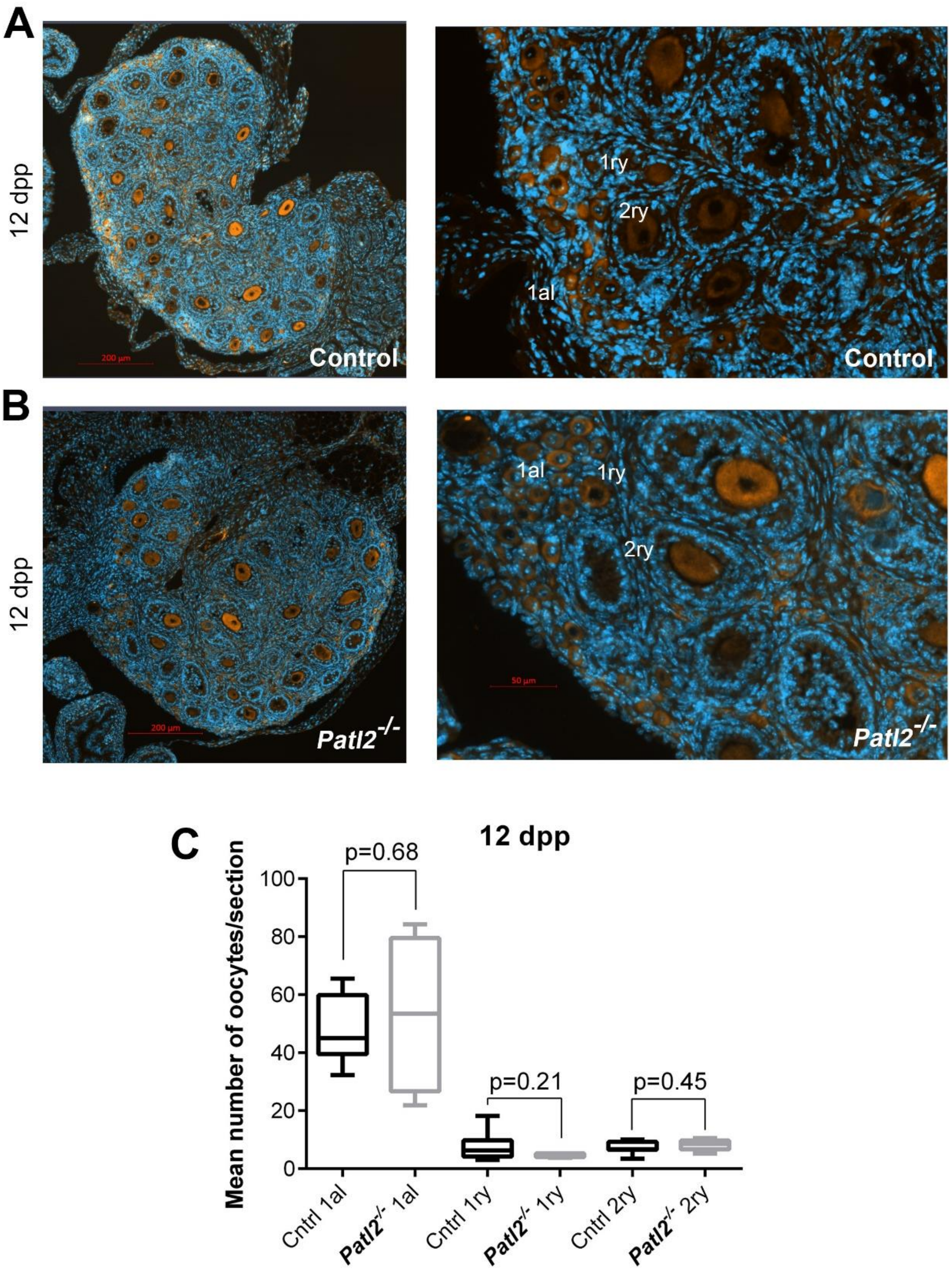

Figure S4 **Ovaries from WT and *Patl2* deficient females have a similar anatomical structure and cellular composition at 12 dpp.**

12-day-old females were euthanised and ovaries were collected and subjected to histological studies by IF. Sections (3 µm thick) were stained with Hoechst to reveal nuclei (blue staining) and an anti-Msy2 antibody (orange staining) to identify oocytes. All oocyte stages were stained by anti-Msy2 **(A)** WT ovaries and **(B)** *Patl2*$^{-/-}$ ovaries. No obvious morphological differences were observed between WT and *Patl2*$^{-/-}$ ovaries. (**C**) The different classes of oocytes (primordial, primary and secondary) were counted in 9 different 3-µm sections from 3 different mice (for each section, 4-7 technical replicates, corresponding to successive sections were counted). n=9, Statistical differences were measured with t-test. P value as indicated.

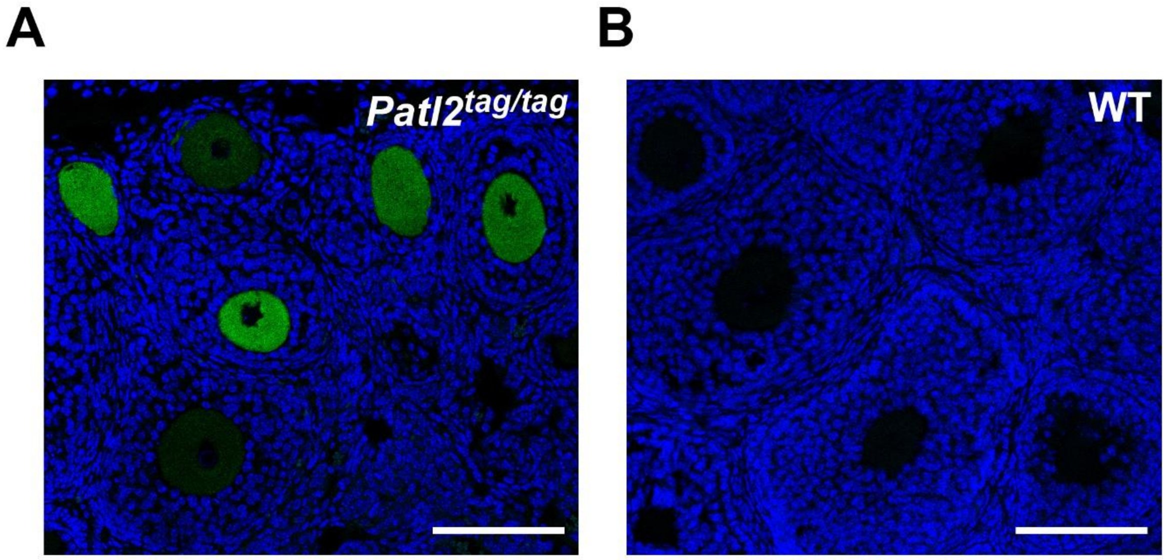

Figure S5. **Validation of the specificity of the HA-antibody in IF experiments**

Comparative fluorescent signals of sections of ovaries from HA-tagged Patl2 (A) and WT (B) mice. Ovaries were stained with Hoechst and an anti-HA tag antibody and observed under confocal microscopy.

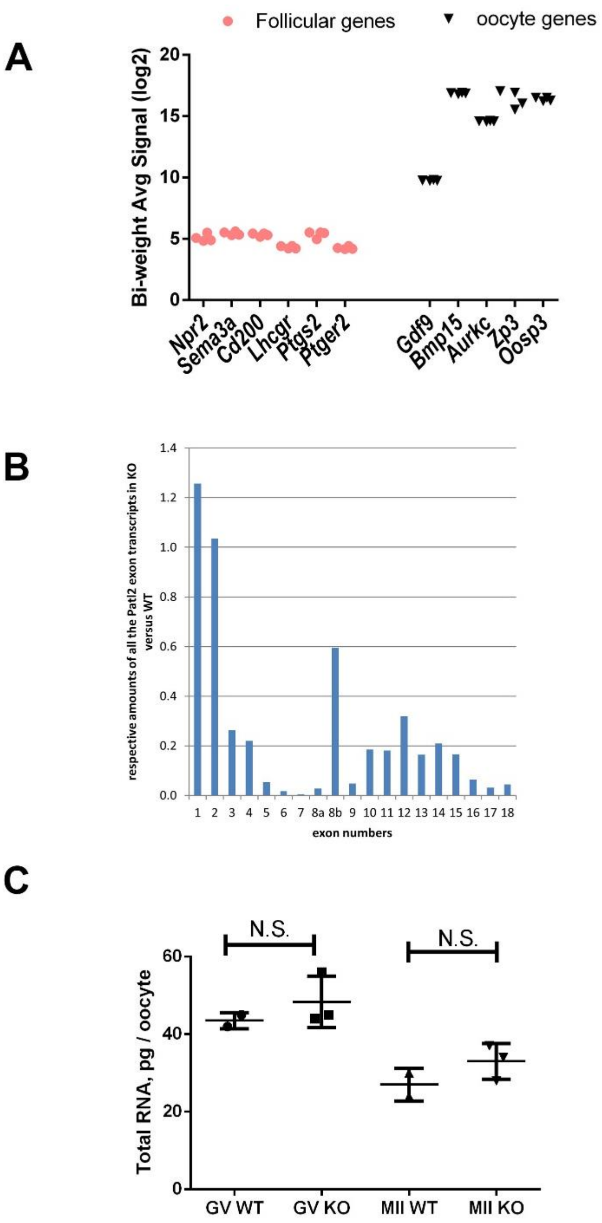

Figure S6. **Characterisation of the RNA sample used in transcriptomic approach.**

(A) Expression levels for specific follicular and oocyte genes. The expression levels for follicle-specific genes were around 5, which is considered a very low level. In contrast, the expression levels for oocyte genes were above 9 and reached the highest values (17). The follicular genes analysed were: Natriuretic peptide receptor 2 (*Npr2*), semaphorin 3A (*Sema3a*), CD200 antigen (C*d200*), luteinizing hormone/choriogonadotropin receptor (*Lhcgr*), prostaglandin G/H synthase 2 (*Ptgs2*) and prostaglandin E receptor 2 (*Ptger*2). The oocyte-specific genes were: growth differentiation factor 9 (*Gdf9*), bone morphogenetic protein 15 (*Bmp15*), aurora kinase C (*Aurkc*), zona pellucida glycoprotein 3 (*Zp3*), oocyte secreted protein 3 (*Oosp3*). **(B)** Comparative expression levels for the different *Patl2* exons. As expected, the level of exon 7 is close to 0, confirming deletion of exon 7 in knock-out females. The expression levels for all 19 Patl2 exons (according to the exon map nomenclature from Affymetrix) were expressed as the ratio (y axis and in linear scale) between exon transcript levels in Patl2$^{-/-}$ oocytes compared to wild type oocytes. In *Patl2*$^{-/-}$ oocytes, *Patl2* exons 1 and 2 were expressed in nearly identical amounts relative to levels measured for wild type oocytes. All the other exons were more or less severely underexpressed or absent. **(C)** Total RNA was extracted from WT and *Patl2*$^{-/-}$ oocytes (both at GV and MII stages) as described in materials and methods. After RT-qPCR assays, the RNA amounts recovered from the purification columns were expressed in pg purified RNA per lysed oocyte. Assuming a consistent RNA recovery yield from the columns, we can conclude that there are non-significant differences in RNA concentrations per oocyte between WT and *Patl2*$^{-/-}$ GV oocytes, as well as between WT and *Patl2*$^{-/-}$ MII oocytes. However, there is a nearly 30 to 40% decrease in the RNA level between MII stage oocytes and GV stage oocytes, as already reported (Su YQ, Sugiura K, Woo Y, et al. Selective degradation of transcripts during meiotic maturation of mouse oocytes. Developmental biology 2007;302:104-17).

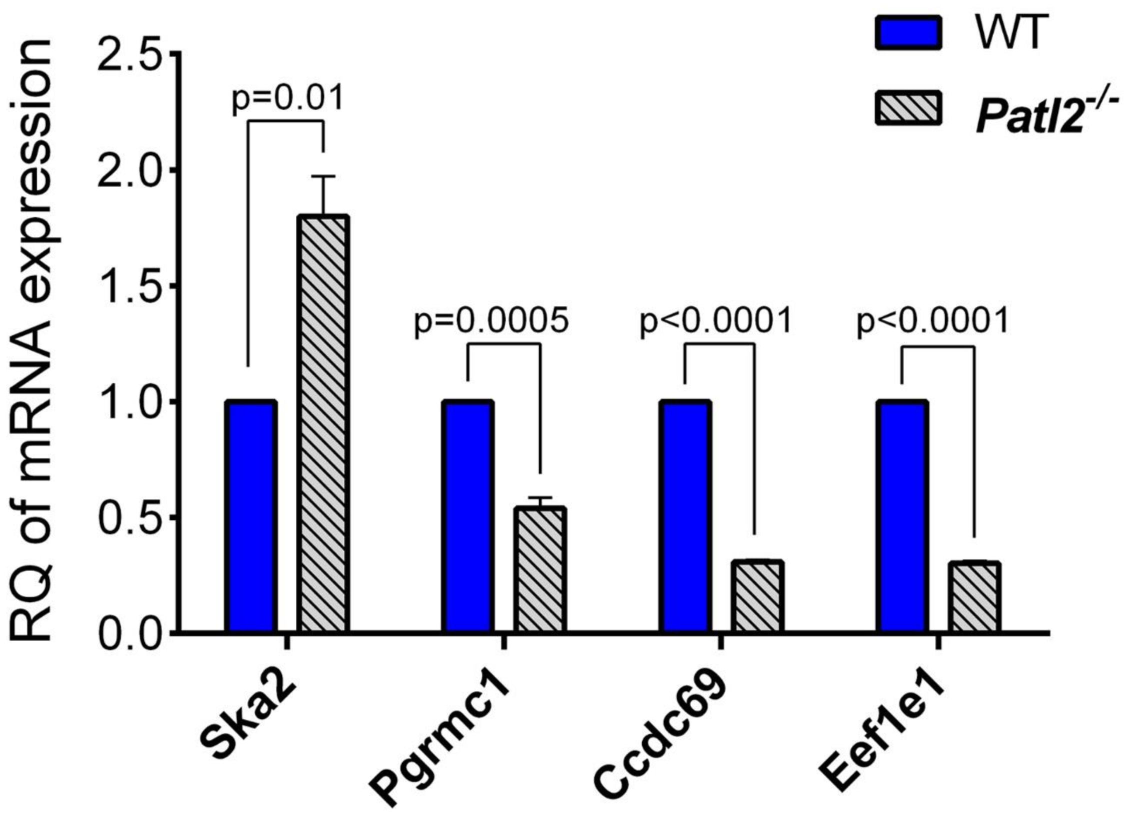

Figure S7. **RT-qPCR experiments validating Affymetrix measures**

Relative quantification (RQ) of 4 genes (*Ska2*, *Pgrmc1*, *Ccdc69* and *Eef1e1*) in germinal vesicle (GV) oocytes from both wild-type (blue) and *Patl2*$^{-/-}$ (grey) mice. n = 8.

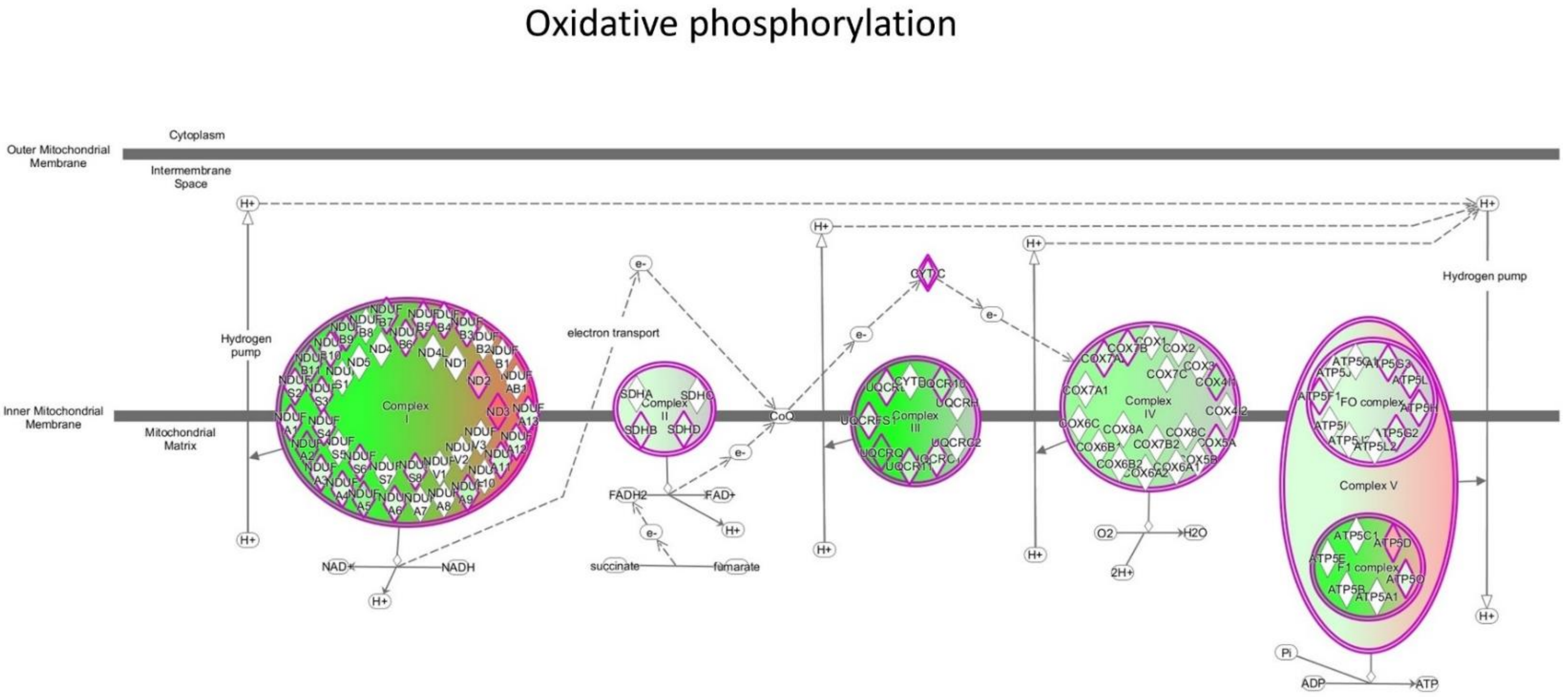

Figure S8. **Deregulated transcripts related to the oxidative phosphorylation pathway**. The interaction network was generated using Ingenuity software and shows that many genes from the oxidative phosphorylation pathway (the major pathway for conversion of energy from NADH oxidation into ATP) are deregulated in both WT and KO samples. Up-regulated transcripts are shown in pink and down-regulated transcripts are indicated in green.

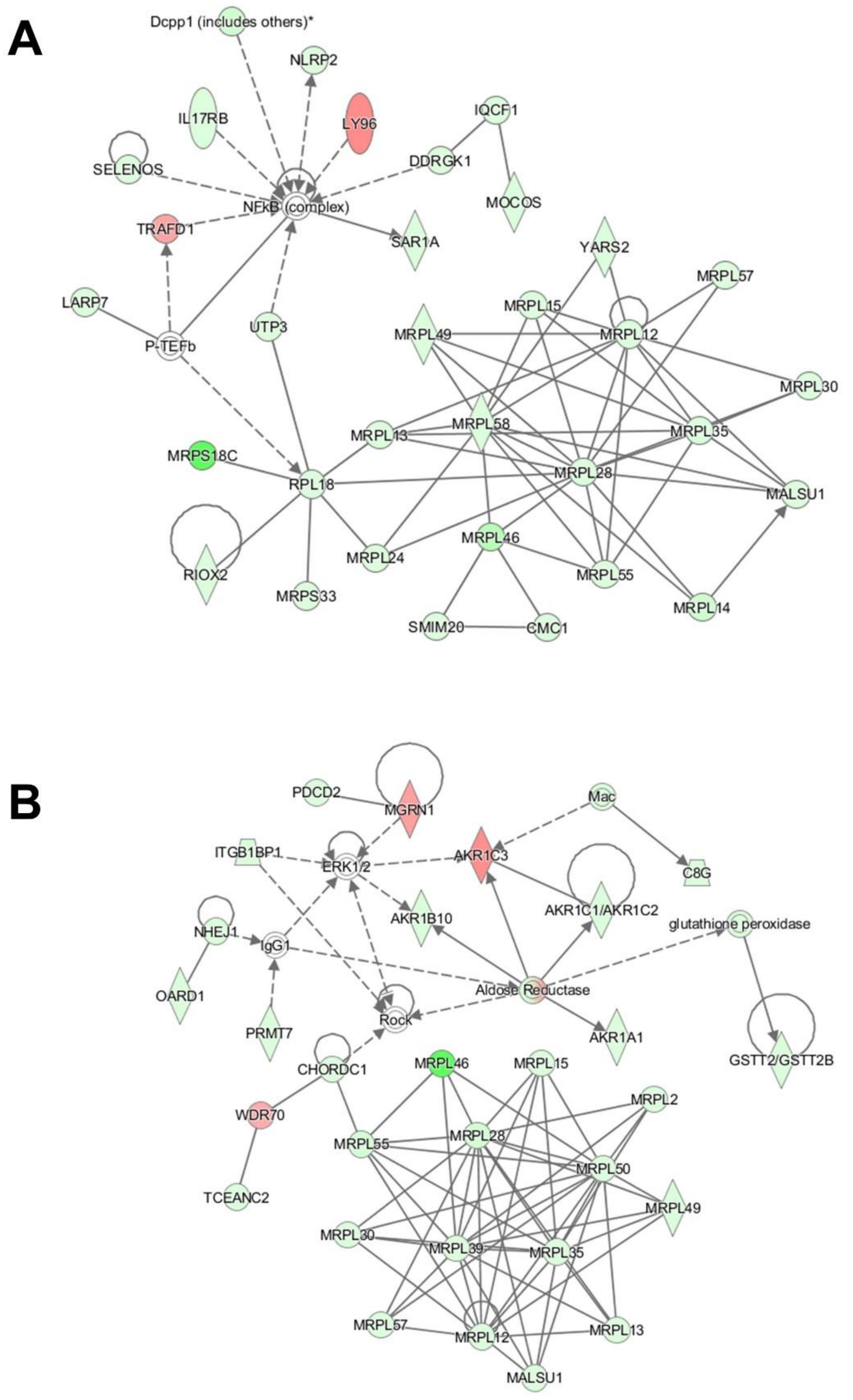

Figure S9. **Functional Network analyses by Ingenuity pathway analysis.** (A) The most significant networks identified in WT included transcripts related to gene expression, protein synthesis, cellular assembly and organisation, and maintenance. (B) The most significant networks in *Patl2*$^{-/-}$ mice assembled transcripts related to gene expression, protein synthesis and lipid metabolism. Up-regulated transcripts are shown in pink and down-regulated transcripts are indicated in green. Continuous lines between nodes indicate direct molecular interactions between connected transcripts; dotted lines indicate indirect functional interactions between transcripts.

SUPPLEMENTARY TABLES

Table S1A. Primer list for Sanger sequencing verification of PATL2 mutation

Oligo name	Sequence	Product size (bp)
PATL2H-Ex6F2	ATGTGCCATGTGGCTGACTT	286
PATL2H-Ex6R2	CAACTGGTCACAAGGGGAGA	

Table S1B. List of primers used to genotype *Patl2 KO* mice and for RT-PCR

	Oligo name	Sequence	Product size (bp)
**Genotyping**	Patl2-Ef	AAGCTCTGTTGGGTTTGAGGAGAAAA	WT allele: 235<br>KO allele: 325
	Patl2-Er	CAGCCTCTTTCCCCTGAATAATTTCA	
	Patl2-Kr	GGGCAAGAACATAAAGTGACCCTCC	
	C-LacZ-F	CCCGTCAGTATCGGCGGAAT	KO allele: 506
	L3R	TGTAATCTGGCTGCAGACAATCTAGGC	
**RT-PCR**	Patl2-RTseq-Ex7F	TCTGTGTTTCCAAGAGCCAGTTC	WT: 135<br>KO: no band
	Patl2-RTseq-Ex8R	GAGCTGGGTCAGATGACTGG	
	βact-F	ACCAGAGGCATACAGGGACA	104
	βact-R	CTAAGGCCAACCGTGAAAAG	

Table S1C. List of primers used to genotype *Patl2-HA tagged* mice and for RT-PCR

Oligo name	sequence	Product size (bp)
**gRNA**	5’-CAAGCAATTAGTTCAGCAGC-3’	
**Patl2_HA-F primer**	5’-CAGACTTTGCCTGGACATCA-3’	351 (WT)<br>378 (KI)
**Patl2_HA-R primer**	5’-GACCATGCTTGGCTCATAG-3’	

Table S1. **List of primers used for mouse genotyping**

Target /Antigen	Species	Supplier	REF	DILUTION
Tubulin	Mouse	Sigma Aldrich	T4026	1/800
MSY2	Mouse	Santa cruz Technology	SC393840	1/200 for IF
HA High Affinity	Rat	Roche/Sigma	11815016001	1/800 for IF, 1/1000 for WB
DDX6	Rabbit	Biotechne	NB200-191	1/500 for IF
CPEB1	Rabbit	Abcam	ab3465	1/200 for IF
Secondary antibody DyLight549 Goat anti mouse	Goat	Jackson Immuno Research	115-505-062	1/800
Secondary antibody AlexaFluor488 Goat anti rabbit	Goat	Jackson Immuno Research	115-545-144	1/800
Secondary antibody AlexaFluor488 Goat anti rat	Goat	Life Technologies	A-11006	1/800
Secondary antibody cy3 (?) Goat anti rabbit	Goat	Jackson Immuno Research	111-165-144	1/400
Secondary antibody HRP conjugate goat anti-rat	Goat	Millipore	AP136P	1/5000

Table S2. **List of antibodies**
