# Supplementary material for: PATL2 is a key actor of oocyte maturation whose invalidation causes infertility in women and mice
Source: EMBO Mol Med. 2018 Apr 16;10(5):e8515. doi: 10.15252/emmm.201708515 (PMC5938616; doi:10.15252/emmm.201708515)
Supplement: Supplementary file 2 — Expanded View Figures PDF [file EMMM-10-e8515-s002.pdf]

Expanded View Figures

Figure EV1. Quality of oocytes collected from patients harbouring *PATL2* mutation and control patients after ovarian stimulation.

A The mean age of the six patients harbouring a *PATL2* mutation at the time of 11 hormonal stimulations was compared to the non-*PATL2* patients within the cohort and also to a control cohort corresponding to women from infertile couples of similar geographical origin where the male was diagnosed with a male infertility (mean ± SEM, n = 234). There was no significant (NS) age difference between the *PATL2* patients, the non-*PATL2* patients and the control cohort.

B Numbers of oocytes retrieved after hormonal stimulation (mean ± SEM) were similar in *PATL2* patients, non-*PATL2* patients and the control cohort.

C Collected oocytes were sorted according to their maturation stage. For patients harbouring *PATL2* mutation, the mean numbers of GV and atretic oocytes were significantly increased and no MII oocytes were collected. Non-*PATL2* patients from the same cohort showed a comparably larger proportion of MI-arrested oocytes.

Data information: Statistical differences were assessed using unpaired two-tailed t-tests.

© 2018 The Authors

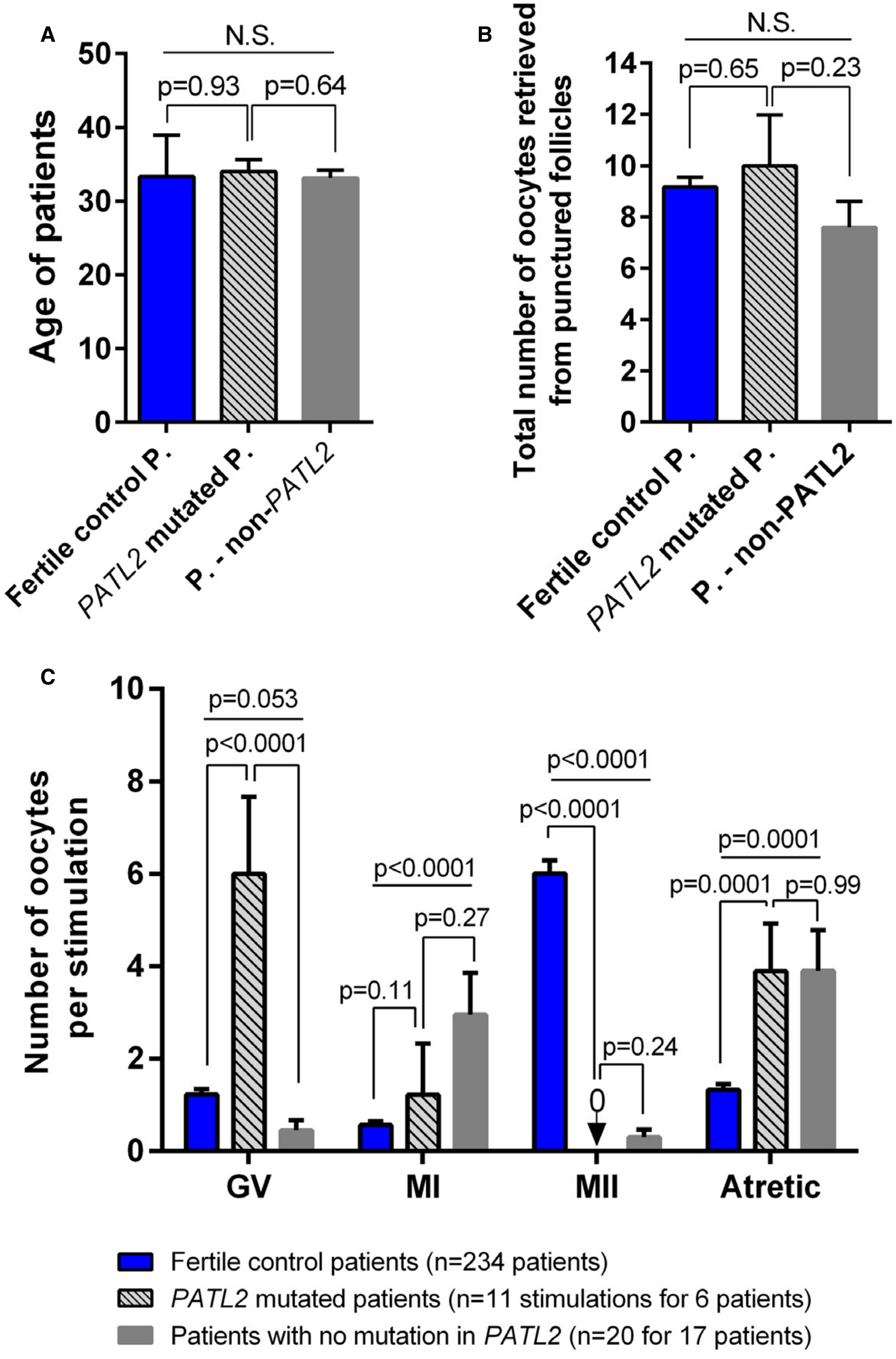

Figure EV1.

© 2018 The Authors

Figure EV2. Histological analysis of ovaries from control and *Patl2*$^{-/-}$ females at 26 days postpartum (dpp).

A Whole section (3 μm thick) of an ovary from a control female at 26 ddp. Section was stained with an antibody against Msy2 (orange staining) to make oocytes clearly visible. Sections were counterstained with Hoechst to reveal the nucleus (blue staining). Right image, corresponding to the enlargement of the red square on the left image, shows the different classes of follicle: primordial (1al), primary (1ry) and secondary (2ry).

B Similar images for an ovary from a *Patl2*$^{-/-}$ female at 26 ddp.

C Comparative numbers of primordial and primary follicle oocytes per section in *Patl2*$^{-/-}$ and control ovaries. Nine different 3-μm sections from three different mice (for each section, four-seven technical replicates corresponding to successive sections were counted). Data are presented on box and whisker plots indicating min. and max. values. Statistical differences were assessed using *t*-test, *P*-value as indicated.

D Comparative number of follicles per section from control and *Patl2*$^{-/-}$ ovaries. Only follicles where oocytes were visible were counted (n = 9 sections per genotype, between four to seven technical replicates). Data are presented on box and whisker plots indicating min. and max. values. Statistical differences were assessed using *t*-test, *P*-value as indicated.

E Comparative amplitude histograms of follicle size per section from control and *Patl2*$^{-/-}$ ovaries (n = 9 sections per genotype with four to seven technical replicates). Statistical differences were assessed using Kolmogorov-Smirnov test.

© 2018 The Authors

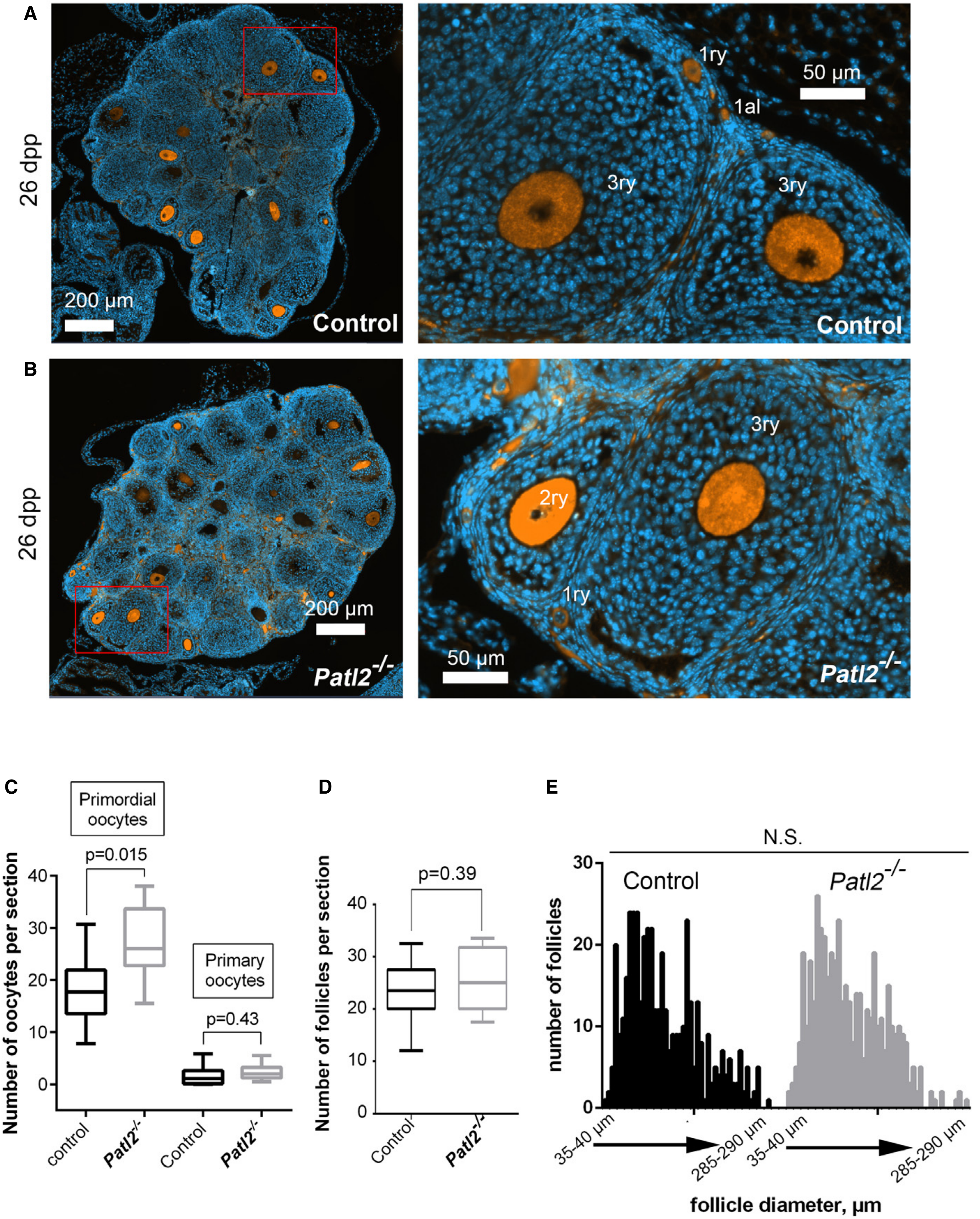

Figure EV2.

 © 2018 The Authors

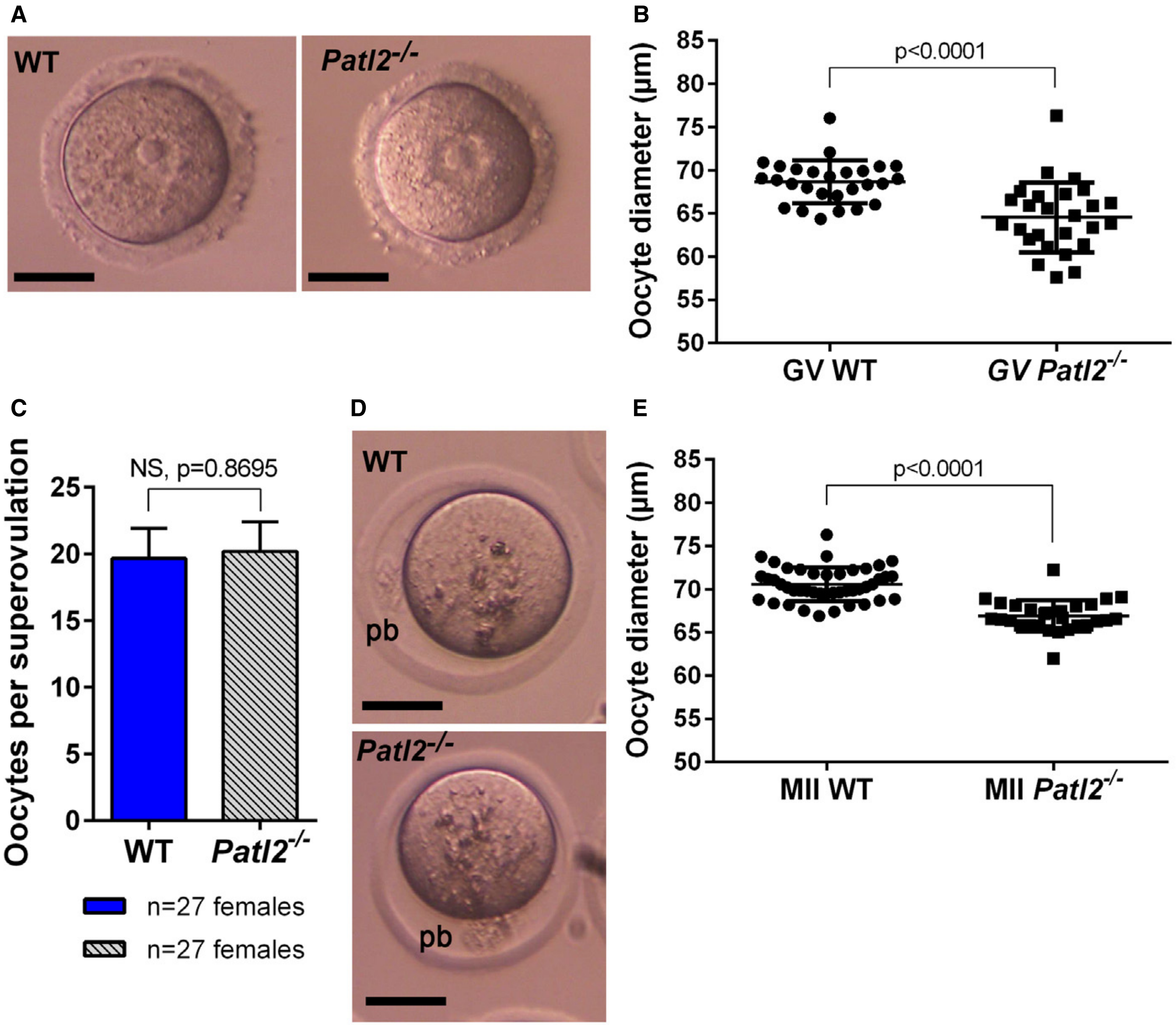

Figure EV3. Lack of Patl2 does not affect number of oocytes produced, but impairs oocyte growth.

A Representative images for a GV oocyte from WT and *Patl2*$^{-/-}$ females stimulated with 5 UI PSMG. Scale bars = 35 μm.

B Comparison of WT and *Patl2*$^{-/-}$ GV oocyte diameter. In the absence of Patl2, the diameter (mean ± SEM) dropped from 68.6 ± 0.5 to 64.5 ± 0.8 μm ($P < 0.0001$). Statistical difference was assessed using unpaired two-tailed *t*-test with Welch's correction.

C The number of oocytes (mean ± SEM) harvested 13 h after full hormonal stimulation (PMSG followed 48 h later by 5 UI HCG) was similar in WT and Patl2-deficient females. Statistical difference was assessed using unpaired two-tailed *t*-test.

D Images of a MII oocytes from WT and *Patl2*$^{-/-}$ females after stimulation (pb = 1st polar body). Scale bars = 35 μm.

E Diameter of WT and *Patl2*$^{-/-}$ MII oocytes (mean ± SEM), the mean drops from 70.6 ± 0.3, n = 44 in the WT to 66.9 ± 0.3, n = 28 in *Patl2*$^{-/-}$ ($P < 0.0001$), oocytes were collected from three females in each case. Statistical difference was assessed using unpaired two-tailed *t*-test.

© 2018 The Authors

Figure EV4. Biological functions and pathways associated with up- or down-regulated differential expression transcripts in *Patl2*$^{-/-}$ versus WT samples at the GV and MII stages.

To investigate possible interactions of up and down-regulated transcripts ($P < 0.05$, absolute fold-change > 2), genes with altered expression profile identified by the Affymetrix microarray were imported into the Ingenuity Pathway Analysis software (IPA) for analysis.

A Canonical pathways identified by IPA that were significantly enriched among transcripts deregulated in *Patl2*$^{-/-}$ GV oocytes with respect to GV-WT oocytes.
B Canonical pathways identified by IPA that were significantly enriched among transcripts deregulated in *Patl2*$^{-/-}$ MII oocytes with respect to MII-WT oocytes.

Data information: *Y*-axis indicates the significance (−log *P*-value) of the functional pathway association, which depends on the number of genes in a class as well as biological relevance. The threshold line represents a *P*-value of 0.05 and was calculated by applying Fischer's test.

© 2018 The Authors

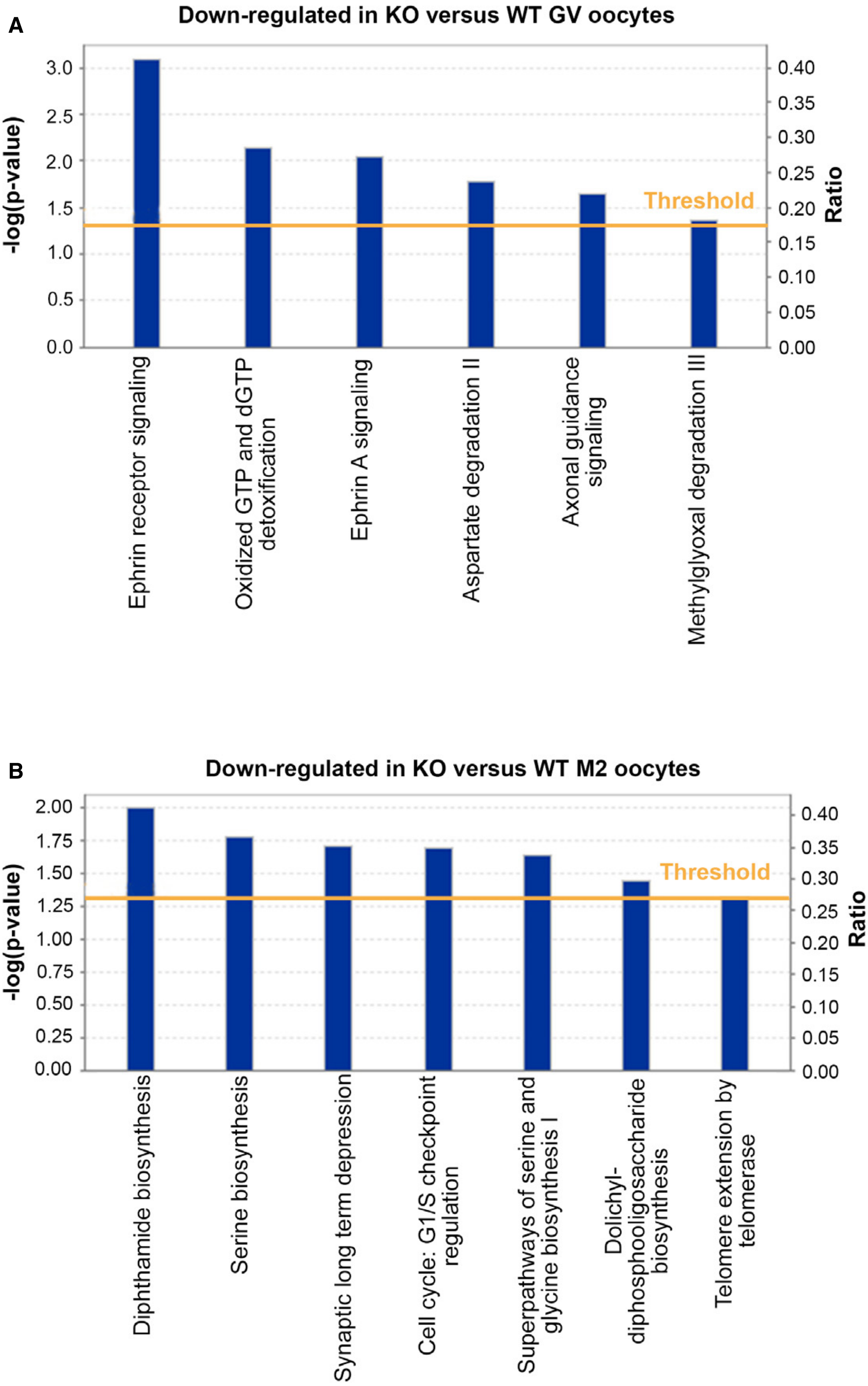
A
Down-regulated in KO versus WT GV oocytes
-log(p-value)
3.0
2.5
2.0
1.5
1.0
0.5
0.0
Ratio
0.40
0.35
0.30
0.25
0.20
0.15
0.10
0.05
0.00
Threshold
Ephrin receptor signaling
Oxidized GTP and dGTP detoxification
Ephrin A signaling
Aspartate degradation II
Axonal guidance signaling
Methylglyoxal degradation III
B
Down-regulated in KO versus WT M2 oocytes
-log(p-value)
2.00
1.75
1.50
1.25
1.00
0.75
0.50
0.25
0.00
Ratio
0.40
0.35
0.30
0.25
0.20
0.15
0.10
0.05
0.00
Threshold
Diphthamide biosynthesis
Serine biosynthesis
Synaptic long term depression
Cell cycle: G1/S checkpoint regulation
Superpathways of serine and glycine biosynthesis I
Dolichyl-diphosphooligosaccharide biosynthesis
Telomere extension by telomerase

Figure EV4.

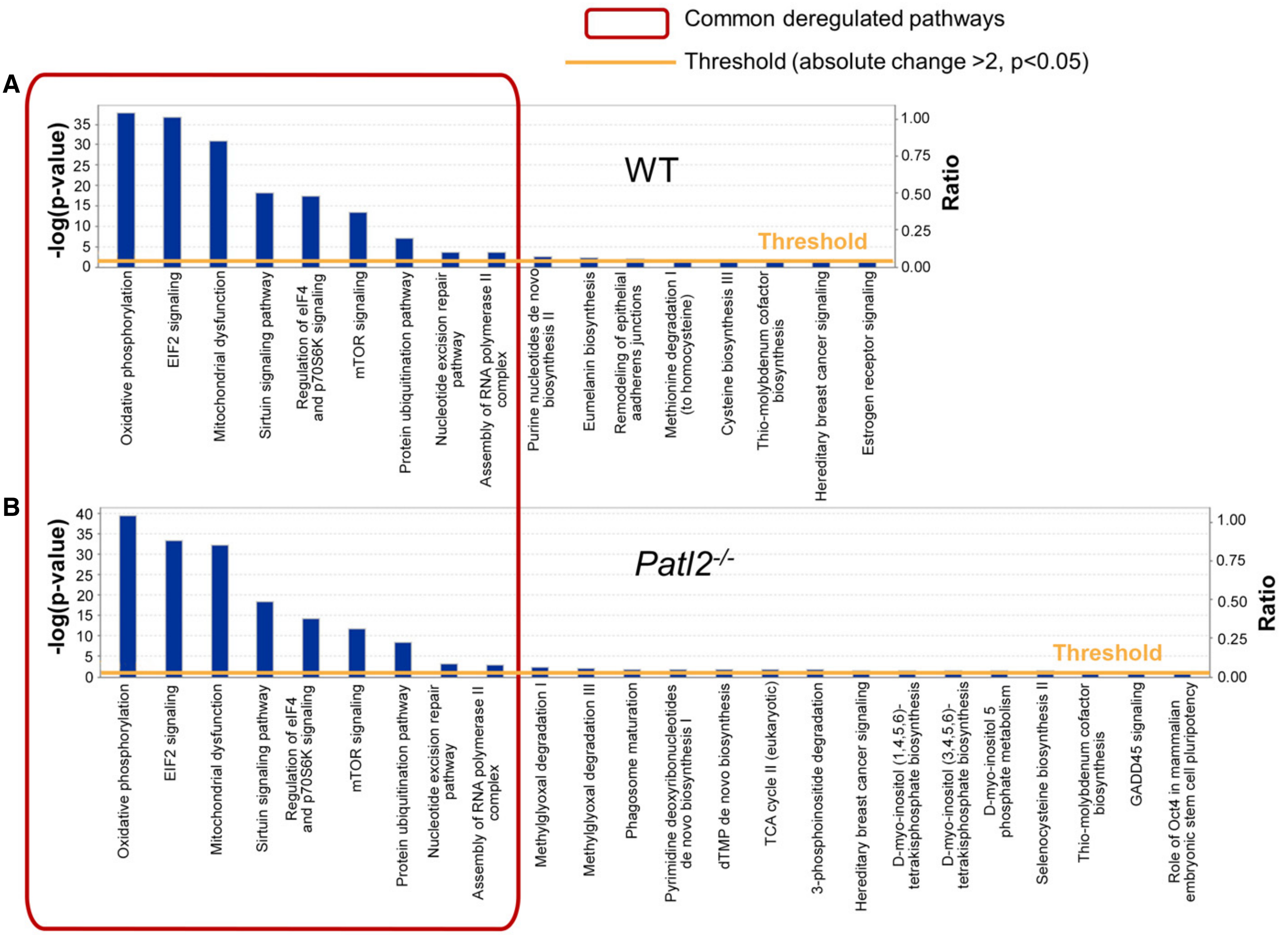

Figure EV5. Transcriptomic analysis of the GV-MII transition. Biological functions and pathways associated with up- or down-regulated differential expression in MII versus GV oocytes from WT and *Patl2*$^{-/-}$ samples.

To investigate possible interactions of up- and down-regulated transcripts ($P < 0.05$, absolute fold-change > 2) between GV and MII stages, genes with an altered expression profile identified by the Affymetrix microarray were imported into the Ingenuity Pathway Analysis software (IPA) for analysis.

A Canonical pathways identified by IPA that were significantly enriched among changed transcripts identified in WT samples at the GV-MII transition.
B Canonical pathways identified by IPA that were significantly enriched among changed transcripts identified in *Patl2*$^{-/-}$ samples at the GV-MII transition.

Data information: *Y*-axis indicates the significance (−log *P*-value) of the functional pathway association, which depends on the number of genes in a class as well as biological relevance. The threshold line represents a *P*-value of 0.05 and was calculated by applying Fischer's test.

© 2018 The Authors
